# Antimicrobial Efficacy of Green Synthesized Nanosilver with Entrapped Cinnamaldehyde against Multi-Drug-Resistant Enteroaggregative *Escherichia coli* in *Galleria mellonella*

**DOI:** 10.3390/pharmaceutics14091924

**Published:** 2022-09-12

**Authors:** Vemula Prasastha Ram, Jyothsna Yasur, Padikkamannil Abishad, Varsha Unni, Diksha Purushottam Gourkhede, Maria Anto Dani Nishanth, Pollumahanti Niveditha, Jess Vergis, Satya Veer Singh Malik, Byrappa Kullaiah, Nitin Vasantrao Kurkure, Chatragadda Ramesh, Laurent Dufossé, Deepak B. Rawool, Sukhadeo B. Barbuddhe

**Affiliations:** 1 Division of Veterinary Public Health, ICAR-Indian Veterinary Research Institute, Izatnagar, Bareilly 243122, India; 2 ICAR-National Research Centre on Meat, Hyderabad 500092, India; 3 Department of Veterinary Public Health, College of Veterinary and Animal Sciences, Pookode, Kerala Veterinary and Animal Sciences University, Pookode 673576, India; 4 Centre for Research and Innovations, BGS Institute of Technology, Adichunchanagiri University, Mandya 571448, India; 5 Department of Veterinary Pathology, Nagpur Veterinary College, Nagpur 440006, India; 6 Biological Oceanography Division (BOD), Council of Scientific and Industrial Research, National Institute of Oceanography (CSIR-NIO), Dona Paula 403004, India; 7 Chemistry and Biotechnology of Natural Products (CHEMBIOPRO Lab), Département Agroalimentaire, Ecole Supérieure d’Ingénieurs Réunion Océan Indien (ESIROI), Université de La Réunion, F-97744 Saint-Denis, France

**Keywords:** antimicrobial resistance, cinnamaldehyde, enteroaggregative *Escherichia coli*, *Galleria mellonella*, silver nanoparticles

## Abstract

The global emergence of antimicrobial resistance (AMR) needs no emphasis. In this study, the in vitro stability, safety, and antimicrobial efficacy of nanosilver-entrapped cinnamaldehyde (AgC) against multi-drug-resistant (MDR) strains of enteroaggregative *Escherichia coli* (EAEC) were investigated. Further, the in vivo antibacterial efficacy of AgC against MDR-EAEC was also assessed in *Galleria mellonella* larval model. In brief, UV-Vis and Fourier transform infrared (FTIR) spectroscopy confirmed effective entrapment of cinnamaldehyde with nanosilver, and the loading efficiency was estimated to be 29.50 ± 0.56%. The AgC was of crystalline form as determined by the X-ray diffractogram with a mono-dispersed spherical morphology of 9.243 ± 1.83 nm in electron microscopy. AgC exhibited a minimum inhibitory concentration (MIC) of 0.008–0.016 mg/mL and a minimum bactericidal concentration (MBC) of 0.008–0.032 mg/mL against MDR- EAEC strains. Furthermore, AgC was stable (high-end temperatures, proteases, cationic salts, pH, and host sera) and tested safe for sheep erythrocytes as well as secondary cell lines (RAW 264.7 and HEp-2) with no negative effects on the commensal gut lactobacilli. in vitro, time-kill assays revealed that MBC levels of AgC could eliminate MDR-EAEC infection in 120 min. In *G. mellonella* larvae, AgC (MBC values) increased survival, decreased MDR-EAEC counts (*p* < 0.001), had an enhanced immunomodulatory effect, and was tested safe to the host. These findings infer that entrapment enhanced the efficacy of cinnamaldehyde and AgNPs, overcoming their limitations when used individually, indicating AgC as a promising alternative antimicrobial candidate. However, further investigation in appropriate animal models is required to declare its application against MDR pathogens.

## 1. Introduction

Globally, antimicrobial resistance (AMR) has been regarded as a looming public health threat. Owing to the indiscriminate use of antimicrobials, the AMR crisis is progressing at a rapid pace among food-borne pathogens, including enteroaggregative *Escherichia coli* (EAEC) [1]. Of late, EAEC has been regarded as one of the key emerging enteric pathogens due to its improved detection in diarrhoeal episodes globally [2]. The emergence of multi-drug resistance (MDR) among the EAEC pathotypes has been reported [3]. The ever-increasing drug-resistant bacterial pathogens reported worldwide cross the human-animal interface by way of close contact, foods (primarily of animal origin), and the associated environment [4]. Hence, coordinated efforts from various sectors (animal, human, and environment) are warranted to regulate the menace of AMR effectively by appropriate therapeutic as well as preventive actions [5], thereby encompassing the core theme of ‘one health’. With the tapered antibiotic discovery pipeline and evolution of AMR among food-borne pathogens, research has recently been focused on the development of alternate therapeutics to combat MDR infections [6]; the use of phytochemicals and nanomaterials is one among them [6,7,8,9].

Since time immemorial, the antimicrobial potential of phytochemical agents has been reported. It has been documented that medicinal plants provide various secondary metabolites as well as essential oils with proven antimicrobial properties [10,11]. For instance, cinnamaldehyde, the major chemical constituent of cinnamon essential oil reported from *Cinnamomum* species, has demonstrated outstanding antimicrobial efficacy against multiple infections [12]. However, these phytochemical compounds are particularly unstable and might get destroyed upon exposure to various physicochemical conditions, such as varying pH, proteases, and cationic salts present in the gastrointestinal tract and high-end temperatures [13]. Moreover, the size and polarity of such agents make them difficult to pass across the mucosa, endothelial lining of blood vessels, gastrointestinal tract, and blood-brain barrier [14]. Therefore, to improve their bioavailability, phytochemical compounds are often combined with suitable nanoparticles to release them at the desired site or target tissue with improved stability and antimicrobial efficacy with minimal toxicity to the host [14,15,16]. Additionally, research suggests that phytochemicals, when coupled with nanocarrier systems, may help delay the development of drug resistance, which ultimately limits the usage of toxic antimicrobials, thereby safeguarding animal, human, and environmental health. Among the nanomaterials, silver nanoparticles (AgNPs) have been widely studied with proven antimicrobial properties against MDR pathogens due to their distinct physicochemical properties [17,18,19]. Although several methods of synthesis of NPs are in place [20], green synthesis is the most extensively practiced mode to reduce their toxicity as well as maintain ergonomic feasibility [21,22,23]. Earlier, we reported the green synthesis of AgNPs with antimicrobial, antifouling, and antioxidant properties using the cell-free supernatant of a potential probiotic *Lactobacillus acidophilus* strain [24]. Furthermore, screening as well as in vivo evaluation of such therapeutic candidates to reveal the host-pathogen interactions against the MDR pathogens is often challenging.

In recent times, *Galleria mellonella* (greater wax moth) larvae have been explored to evaluate the therapeutic potential of novel candidates against various bacterial pathogens, including MDR-EAEC strains [25,26,27]. With the short life span of larvae and their ability to simulate humans while exploring pathogens of public health significance [28,29,30], these insect larvae remain outstanding in vivo models to screen novel therapeutics. Even though the in vitro antimicrobial efficacy of cinnamaldehyde as well as AgNPs has been reported against a variety of MDR pathogens [31,32,33], the in vitro as well as in vivo efficacy studies of nanoparticle-entrapped phytochemicals appear to be scanty. The objective of the present study was to entrap cinnamaldehyde with the synthesized nanosilver and then evaluate its antimicrobial potential against the field strains of MDR-EAEC strains. Initially, the in vitro stability, safety, and antimicrobial efficacy of nanosilver-entrapped cinnamaldehyde (AgC) were evaluated against MDR-EAEC; finally, the in vivo efficacy of AgC against MDR-EAEC strains in a *G. mellonella* larval model was explored using appropriate controls for its possible utility as an effective therapeutic candidate.

## 2. Materials and Methods

### 2.1. Bacterial Strains, Phytochemicals, and Nanoparticles

Three characterized field isolates of MDR enteroaggregative *E. coli* designated as MDR-1 (NCBI GenBank: KY941936.1); MDR-2 (NCBI GenBank: KY941937.1), and MDR-3 (NCBI GenBank: KY941938.1) maintained in the laboratory repository were re-validated by PCR assay [34] prior to their inclusion in the study. For antimicrobial susceptibility testing, *E. coli* ATCC 25922 served as the quality control strain. 

The AgNPs were synthesized using the cell-free supernatant of a potential probiotic strain, *Lactobacillus acidophilus* MTCC 10307 [24]. Cinnamaldehyde (≥95% purity; Sigma Aldrich, St. Louis, MO, USA) identified from our earlier study [31] was employed for entrapment with AgNPs.

### 2.2. Entrapment of AgNPs with Cinnamaldehyde

The characterized AgNPs were entrapped with cinnamaldehyde (AgC) to produce a product that has both properties, as described earlier, with certain modifications [35]. In brief, the entrapment procedure was optimised by adding cinnamaldehyde gently to the synthesized AgNPs at a 1:10 ratio with continuous stirring at room temperature for 24–48 h. Subsequently, the entrapped mixture was washed thrice at 10,000× *g* for 20 min to collect the pellet, which was finally washed in methanol (Loba Chemie, Mumbai, India) and then freeze-dried to collect the powder. 

The loading capacity (LC) of AgC (in %) was estimated using the following equation: LC = (Total amount of cinnamaldehyde loaded (in mg)/weight of AgNPs after drying (in mg)) × 100 [36].

### 2.3. Characterization of AgC

The entrapped AgC was monitored by scanning within the range of 250 to 700 nm using a UV-visible spectrophotometer (ThermoFisher Scientific, Waltham, MA, USA), keeping AgNPs and cinnamaldehyde as suitable controls. To determine the chemical functional groups on AgC, Fourier transform infrared (FTIR) spectroscopy (Perkin Elmer C94012, Akron, OH, USA) was performed, and the spectra were recorded within the range of 400 to 4000 cm^−1^, at a resolution of 4 cm^−1^. The structural investigations of AgC were carried out with a powder X-ray diffractometer (PXRD; Bruker D8 Advance, San Jose, CA, USA) operated at CuKα radiation, 40 keV, and 40 mA using radiation with a scanning step size of 0.02° (ƛ = 1.54060 Å). The average size of AgC was estimated by using Debye–Scherrer’s equation, L= (kλ)/(βcosθ), where k is the Scherrer constant, λ indicates X-ray wavelength, β is the full width at half maximum (FWHM) of the measured reflection, and θ is the angle of diffraction. Further, the morphological investigations of AgC were carried out by scanning electron microscopy (SEM; Jeol 6390LV, Tokyo, Japan) at different magnifications, while transmission electron microscopy (TEM; JEM 2100, Jeol, Tokyo, Japan) was employed to analyse the morphology as well as the size of AgC.

### 2.4. Determination of Minimum Inhibitory Concentration (MIC) and Minimum Bactericidal Concentration (MBC)

The MIC and MBC values of AgC against the MDR-EAEC strains, as a measure of antimicrobial efficacy, were determined by the micro broth dilution method [37].

The MIC was determined by incubating 50 μL of the individual test cultures of MDR-EAEC (1 × 10^7^ CFU/mL) in cation-adjusted Mueller–Hinton (CA-MH; HiMedia, Mumbai, India) broth medium (50 μL) with decreasing concentrations of treatment compounds (0.1024–0.000025 mg/mL) in 96-well flat-bottom microtiter plates for 24 h, where each plate included a positive control (1 × 10^7^ CFU/mL test culture) and a negative control (CA-MH broth control). The lowest concentration of compound without visible growth was designated as the MIC. For MBC determination, approximately 10 μL of the seeded inoculum was drawn from each well, having no visible growth, and placed on CA-MH agar (HiMedia, Mumbai, India). The lowest concentration that produced 99.90% killing of the test culture was considered the MBC value of the compound [38,39].

### 2.5. In Vitro Stability Assays

The in vitro stability of AgC was evaluated by exposing it to high-end temperatures (70 °C and 90 °C), varied pH concentrations (4.0, 6.0, and 8.0), physiological concentrations of cationic salts (150 mM NaCl and 2 mM MgCl_2_), protease enzymes (trypsin, proteinase-K, and lysozyme), and sera (sheep and poultry) in comparison with cinnamaldehyde and AgNPs, keeping appropriate controls [39].

The thermostability of treatment compounds (cinnamaldehyde, AgNPs, and AgC) was determined by individually treating them at 70 °C and 90 °C for 5, 15, and 30 min, then measuring the MIC value, while keeping an untreated treatment at room temperature as a control. 

Individual compounds were exposed to different pH concentrations (pH 4, 6, and 8) in adjusted CA-MH broth, followed by inoculation with MDR-EAEC (n = 3), then estimating the MIC and MBC values as previously described. CA-MH broth adjusted at respective pHs and inoculated with corresponding strains of MDR-EAEC served as control.

The stability of treatment compounds in the presence of physiological concentrations of cationic salts was evaluated by co-incubating the treatment compounds with NaCl (150 mM) and MgCl_2_ (2 mM) in cation-adjusted Mueller Hinton (CA-MH) broth containing the MDR-EAEC strains and then determining the MIC and MBC values keeping untreated treatment controls. 

The effect of protease enzymes (trypsin, proteinase-K, and lysozyme) on the antimicrobial activity of treatment compounds (protease:treatment compound—1:100 (*w/w*)) under study was investigated by incubating the individual compound with each of the enzymes at 37 °C for 5, 15 and 30 min. In order to rule out the antimicrobial activity of the proteases, each protease was used alone in the corresponding buffer as a control. The antimicrobial activity of enzyme-treated compounds against MDR-EAEC strains was determined by measuring the MIC and MBC values after they were exposed to 90 °C for 10 min to inactivate the residual protease activity. 

The stability of the compounds was investigated against sheep and poultry serum keeping appropriate control. The treatment compounds and sera were co-incubated in the assay at 37 °C in a ratio of 1:100 (*w/w*) and heated at 90 °C for 10 min at different time intervals (5, 15, and 30 min) to inactivate the serum activity before determining the MIC and MBC values.

### 2.6. In Vitro Safety Assays

A haemolytic assay employing sheep RBCs and secondary cell line (murine macrophage RAW 264.7 and human epithelioma HEp-2)-based MTT cytotoxicity assay was carried out to ascertain the in vitro safety of AgC (1X, 2X, 4X, and 10X MIC). Moreover, the AgC at different concentrations (1X and 2X) was also tested to investigate the adverse effect, if any, on the beneficial strains of gut lactobacilli (*L. acidophilus* MTCC 10307 and *L. plantarum* MTCC 5690) [38,39].

In brief, the haemolytic activity of the compounds tested was determined by measuring the haemoglobin release from sheep erythrocytes at 540 nm. The percentage of haemolysis was calculated as (A_Sample_—A_PBS_)/(A_Triton-X_—A_PBS_) × 100, wherein A_Sample_ is the absorbance of treated cinnamaldehyde/AgNPs/AgC, A_PBS_ is the absorbance of untreated control with PBS, and A_Triton-X_ is the absorbance of lysed cells treated with Triton X- 100 measured at 540 nm. Furthermore, the safety of cinnamaldehyde, AgNPs, and AgC (1X, 2X, 4X, and 10X MIC) was assessed using the MTT [3-(4,5-dimethylthiazole-2-yl)-2,5-diphenyl tetrazolium bromide] assay on secondary cell lines, namely, human epithelioma cell line (HEp-2) and murine macrophage cell line (RAW 264.7).

The treatment compounds were also tested at different concentrations (1X and 2X MIC) to see if they had any inhibitory effects on beneficial gut lactobacilli (*L. acidophilus* MTCC 10307 and *L. plantarum* MTCC 5690) strains. In brief, Mann–Rogosa–Sharpe (MRS) broth medium (100 µL) containing treatment compounds (1X and 2X MIC) was inoculated with 100 µL of each beneficial gut lactobacilli (*L. acidophilus* and *L. plantarum* ca. 1 × 10^7^ CFU/mL) in 96-well microtiter plates. A positive growth control (untreated commensal flora) and negative growth control (MRS broth) were included on each plate. After incubation at 37 °C for 48 h, the antibacterial effect of cinnamaldehyde, AgNPs and AgC on commensal lactobacilli was measured by observing the absorbance at 600 nm (Thermo Scientific Multiskan GO) as well as drawing two 10 µL samples from each well and plating them onto MRS agar plates.

### 2.7. In Vitro Time- and Dose-Dependent Time-Kill Assay

The in vitro time- and dose-dependent growth kinetics of MDR-EAEC isolates (n = 3) were evaluated by co-incubating the log-phase cultures of each MDR-EAEC isolate (1 × 10^7^ CFU/mL) in cation-adjusted Mueller–Hinton (CA-MH) broth treated with 1X MBC concentration of AgC in triplicate (Supplementary Section S1). The respective MDR-EAEC isolates (n = 3) in CA-MH broth served as untreated controls, whereas the MDR-EAEC isolates treated with meropenem (10 μg/mL) were used as the treatment controls. After incubation at 37 °C, the aliquots were drawn at fixed time intervals (0, 30, 60, 90, 120, 150, and 180 min) to enumerate the MDR-EAEC counts, expressed as log_10_CFU/mL.

### 2.8. In Vivo Antibacterial Efficacy of AgC in the G. mellonella Larval Model

The assessment of the in vivo antimicrobial efficacy of AgC against the MDR strains of EAEC was carried out in the final instar stage of *G. mellonella* larvae [25]. The larvae (about 200–250 mg) were injected with aliquots of MDR-EAEC suspensions (10 µL) via the last right pro-leg using a Hamilton syringe (26 gauge) and monitored at 37 °C in a dark environment. The larvae were maintained in a sterile environment and fed *ad libitum*. The LD_50_ dose of MDR-EAEC strains determined in the larvae [25] was verified and used further to assess the antibacterial activity. 

A total of 11 groups, each comprising 40 *G. mellonella* larvae, were formed: group I (MDR-EAEC infection control), groups II to V (infection groups treated with cinnamaldehyde, AgNPs, AgC, and meropenem), groups VI to IX (cinnamaldehyde, AgNPs, AgC and meropenem as treatment controls), group X (PBS control) and group XI (healthy control). The LD_50_ dose of MDR-EAEC was administered to the larval groups I to V, while groups II to V received an MBC dose of the respective treatment 1 h post-treatment (p.t.). Meanwhile, the larval groups VI to IX were administered with an MBC dose of respective treatment alone, and group X was injected with sterile PBS.

The survival rate, MDR-EAEC counts, melanization rate, hemocyte density, and lactate dehydrogenase (LDH) cytotoxicity assay (Supplementary Sections S2–S5) of the larval groups were subsequently studied at 6 h intervals up to 24 h, followed by 24 h intervals up to 120 h p.t. 

### 2.9. Statistical Analysis

All the experiments were carried out three independent times in triplicate. The results are presented as mean ± standard deviation for each assay and were analysed statistically using GraphPad Prism 5.01 software (GraphPad Software Inc., San Diego, CA, USA). A one-way analysis of variance (ANOVA) with Bonferroni multiple comparison post-test was used to compare the differences observed in the in vitro cell line-based cytotoxicity assay, while a paired two-tailed t-test was used to determine the effect of AgC on commensal gut lactobacilli. The in vitro time-kill assay as well as in vivo time-dependent antimicrobial assays were analysed using a two-way (repeated measures) ANOVA with Bonferroni multiple comparison post-test. The LD_50_ dose of MDR-EAEC strains was determined using the probit regression model, whereas the in vivo *G. mellonella* larval survival curves were determined using the log-rank (Mantel-Cox) test and the log-rank test for trends. A *p*-value of ≤0.01 was considered highly significant, while a *p*-value ≤ 0.05 was considered statistically significant.

## 3. Results and Discussion

The discovery of antibiotics has revolutionised the field of modern medicine and has widely been administered for the treatment of a multitude of bacterial infections in human and veterinary practice. Nonetheless, indiscriminate use of antibiotics and selection pressure have resulted in the emergence of drug-resistant bacterial strains in healthcare settings [1]. EAEC, a potential food-borne bacterial strain that crosses species boundaries, is responsible for chronic as well as persistent diarrhoea, leading to the destruction of the intestinal epithelium [40]. The pathogen is reported to adhere to the intestinal mucosa by aggregative adherent fimbriae (AAF) and form biofilms, as evidenced by the stacked-brick pattern on HEp-2 cells, which is linked to the persistence of infection and, in turn, drug resistance [27,40]. EAEC produces a variety of toxins (enterotoxins and cytotoxins), resulting in inflammatory responses, secretory diarrhoea, and mucosal cytotoxicity. Of late, the emergence of MDR-EAEC strains has been reported at a faster rate than previously thought [41,42,43,44,45,46], and this necessitates alternative therapeutic strategies to combat the drug-resistant strains. 

The EAEC strains (n = 3) used in this study were found to be resistant to four or more classes of antibiotics (Supplementary Table S1) and hence were designated as MDR-EAEC. In recent times, alternative therapeutic approaches such as specific antibodies, phages, exolysins, endolysins, vaccines, probiotics, cationic peptides, phytochemicals, and nanoparticles have been investigated [6,27,47,48]. Studies have been reported to assess the antibacterial activity of such compounds alone; however, investigations employing combinatorial approaches have seldom been documented. Although these compounds have widely been used as antibacterial agents against a variety of MDR pathogens [10,18,19], studies demonstrating antibacterial efficacy against MDR-EAEC strains are lacking. Nanotechnological interventions aim at the delivery of active agents with the intention of targeted delivery, reduction in toxicity with non-compromised safety, improved stability, and therapeutic efficacy with improved bioavailability by minimizing the concentration of the drug [49]. In the present study, in vitro as well as in vivo antibacterial efficacies of a phytochemical compound (cinnamaldehyde) entrapped with the AgNPs were evaluated against MDR-EAEC strains. Owing to the unstable bonds and aldehyde group in its chemical structure, cinnamaldehyde can be oxidised when exposed to air at ambient temperature during manufacture and storage and may become unstable [13]. As a result, when the cinnamaldehyde was entrapped with AgNPs, it appeared to be more stable and consistent over time. In this study, a better loading capacity of 29.50 ± 0.56% was observed in the AgC at the ratio of 1:10 and was employed further throughout this study.

### 3.1. Characterization of AgC

**Figure 1:**
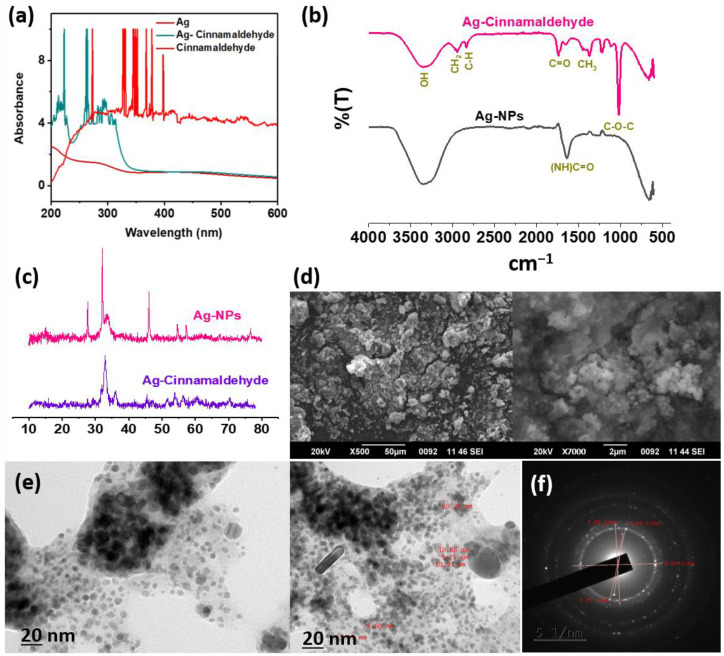
Physico-chemical characterisation of nanosilver-entrapped cinnamaldehyde. UV-Vis spectroscopy (**a**), FTIR spectra (**b**), XRD pattern (**c**), SEM imaging (**d**), TEM imaging (**e**), and SAED pattern (**f**) of AgC.

UV-Vis spectroscopy was used as the most fundamental method for characterizing the nanoparticles. In this study, AgNPs and cinnamaldehyde showed a progressive surface plasmon resonance peak at 430 nm and 290 nm, respectively [24,50]. The peaks displayed by the AgC corresponded to the individual peaks of its components, indicating entrapment (Figure 1a).

The FTIR analysis of the AgC was carried out to detect various functional groups formed after the entrapment of cinnamaldehyde with AgNPs that would be responsible for biological activities. Apart from the characteristic peaks of AgNPs observed at 3350 cm^−1^, 1635 cm^−1^, 1240 cm^−1^, 1170 cm^−1^, and 650 cm^−1^ in the FTIR spectra [24], several other peaks were observed in the entrapped compound (Figure 1b). The peak at 2830 cm^−1^ (C–H stretch) belonged to the alkane group, while the peaks noted at 2940 cm^−1^, 1400 cm^−1^, and 1020 cm^−1^ denoted the presence of CH_2_, CH_3_, and C–O–C groups, respectively. The appearance of these peaks indicated that the functional groups of the cinnamaldehyde had covered the surface of nanoparticles. The FTIR results clearly showed the presence of cinnamaldehyde-related bonds that were likely involved in the entrapped AgC, as well as the possibility that these compounds played an important role in the stabilization of AgC by capping and preventing agglomeration, thereby facilitating enhanced stability and antimicrobial efficacy. Thus, the results of the FTIR analysis confirmed the entrapment of cinnamaldehyde with nanosilver. 

Later, the crystallinity and phase variety of the AgC were determined by PXRD analysis (Figure 1c). The peaks (2θ) observed in the PXRD pattern of AgC at 27.8°, 32.2°, 46.2°, and 76.7° corresponded to the lattice planes (98), (101), (200), and (311), respectively [51]. The X-ray diffractogram of the nanosilver-entrapped cinnamaldehyde revealed a face-centered cubic structure, which correlated with the standard powder diffraction card of the Joint Committee on Powder Diffraction Standards (JCPDS Card No. 00-004-0783) [51,52,53]. The sharp and strong peaks of AgC observed in the XRD pattern revealed that it had a crystalline structure. Moreover, the high peaks observed in the XRD pattern of AgC corresponded to the presence of nanosilver. Furthermore, the expansion of Bragg’s peaks at the bases indicated the production of small-sized AgNPs, whereas the entrapment of cinnamaldehyde on AgNPs corresponded to the unassigned peaks [51]. In this study, an average size of 18.53 ± 1.482 nm was estimated for AgC from the PXRD analysis by using Debye–Scherrer’s equation. Further, the XRD data revealed no major change in the structure of the nanoparticles on entrapment with cinnamaldehyde, i.e., the crystalline nature of the AgNPs was retained even after entrapment. 

SEM was used to examine the surface morphology and shape of the AgC, and the micrographs revealed spherical aggregated forms (Figure 1d). Secondary metabolites found in phytocompounds may explain the aggregation observed in SEM imaging [51]. Moreover, the morphology of AgC was found to be mono-dispersed spherical, as evidenced by TEM imaging. Furthermore, the average size of the AgC estimated from the TEM images was found to be 9.243 ± 1.83 nm (Figure 1e), while the SAED pattern confirmed the crystalline nature of AgC (Figure 1f) and correlated with the XRD analysis. Since agglomeration of NPs could limit antimicrobial efficacy due to the decrease in surface area, which also affected its interaction with bacteria, minimal agglomeration observed in this study for AgC would indicate potential antibacterial activity [54].

### 3.2. Determination of MIC and MBC

As an antibacterial indicator, the MIC and MBC values of AgC were determined against MDR-EAEC strains. The MIC values of the pure phytocompound (cinnamaldehyde) tested ranged from 0.512 to 0.256 mg/mL, and those of AgNPs and AgC tested against MDR-EAEC isolates were in the range of 0.008–0.016 mg/mL. However, the MBC values were found to be either equal to or twice as high as the MIC values, with isolate-specific variations (Table 1). This variation in MIC and MBC values could be attributed to bacterial strain differences, variations in virulence factors, or structural differences in the bacterial cell membrane [31].

In this study, AgC exhibited strong antimicrobial properties against MDR-EAEC strains, which could be due to the combined action of both AgNPs and cinnamaldehyde. Earlier studies reported cinnamaldehyde to have strong antibacterial activity targeting bacterial cell membranes, resulting in the loss of cellular components [31,55]. AgNPs have been reported to act against bacteria by producing reactive oxygen species, releasing silver ions from AgNPs that bind to the bacterial cell membrane, causing protein denaturation by bonding with sulfhydryl groups or attaching AgNPs to bacteria, causing bacterial cell damage [56,57]. Thus, exposing EAEC cells to the MIC levels of AgC would alter their morphology, membrane integrity, and permeability.

### 3.3. In Vitro Stability Assays

The parameters influencing AgC interactions with biological systems were investigated. AgC was subjected to in vitro stability assays (high-end temperatures, pH, cationic salts, protease, and serum treatment). In this study, all the compounds tested (AgC, cinnamaldehyde, and AgNPs) retained their antimicrobial activities even after subjecting to varied stability conditions for MDR-EAEC strains.

When cinnamaldehyde was exposed to high-end temperatures (70 °C and 90 °C), the MIC and MBC values were either equal or increased two-fold. However, the antibacterial activity of cinnamaldehyde after entrapment (AgC) against MDR-EAEC strains was found to be stable over the time intervals, even after exposure to high-end temperatures, indicating that they were thermostable, especially during animal feed preparation or pelleting practices [39] (Supplementary Table S2A). 

Cinnamaldehyde exhibited similar or two-fold higher MIC and MBC values against MDR-EAEC test strains when exposed to different pH conditions (4.0, 6.0, and 8.0). Despite this, when treated at pH 4.0, all of the MDR-EAEC test strains showed a reduction in both the MIC (6-fold) and MBC (8-fold) values of AgC. Surprisingly, AgC retained its antimicrobial activity when treated at higher pH (6.0 and 8.0), indicating pH stability (MIC and MBC values) (Supplementary Table S2B). 

The antimicrobial activity of AgC was tested after the addition of physiological concentrations of different cationic salts (150 mM NaCl and 2 mM MgCl_2_) to investigate salt sensitivity. Even after co-incubating with the cationic salts, the MIC and MBC values of the compounds remained unaltered, indicating that they were unaffected by cationic salts (Supplementary Table S2C). Both the MIC and MBC values of cinnamaldehyde increased up to 2-fold when exposed to protease enzymes (trypsin, proteinase-K, and lysozyme) for different time intervals. Regardless of the MDR-EAEC strains tested, when AgC was co-incubated with the proteases (trypsin, proteinase-K, and lysozyme), the MIC and MBC values remained the same, indicating stability against proteases (Supplementary Table S2D).

In addition, the stability of AgC was also determined using sheep and poultry sera, as the presence of enzymes and proteins in the serum alters its stability. The MIC and MBC values of AgNPs and AgC remained unchanged after co-incubation with sheep and poultry sera at different time intervals against the MDR-EAEC test strains. With cinnamaldehyde, however, a two-fold increase in the MIC and MBC values was observed at various time intervals against all MDR-EAEC test strains (Supplementary Table S2E). In short, the AgC was found to be stable under a variety of physico-chemical conditions (pH, temperatures, cationic salts, sera, and proteases).

### 3.4. In Vitro Safety Assays

The nanoparticles may be translocated to the target cells, tissues, and organs via systemic circulation; therefore, they need to be assessed for their safety. In this context, toxicity studies using RBCs and secondary cell lines are commonly employed. RBCs are thought to be excellent osmometers since every change in the osmotic, as well as physical circumstances, results in haemolysis [24,58]. Therefore, a sheep RBC-based haemolytic assay was employed to ensure the safety profile of AgC (Table 2), wherein concentration-dependent haemolysis was observed with AgC. Interestingly, minimal haemolysis (less than 5%) was observed with AgC at all the levels of MIC (1X, 2X, 5X, and 10X). However, at higher MIC levels (5X and 10X), moderate haemolysis (10–12%) was observed with cinnamaldehyde. When nanoparticles interact with RBCs, their size, along with their structure, shape, and surface coating, is one of the most important factors in determining their toxicity and absorption efficiency [59]. As a result of the findings, AgC was found to have less haemolytic activity than cinnamaldehyde and AgNPs alone and thus could be considered haemocompatible.

**Figure 2:**
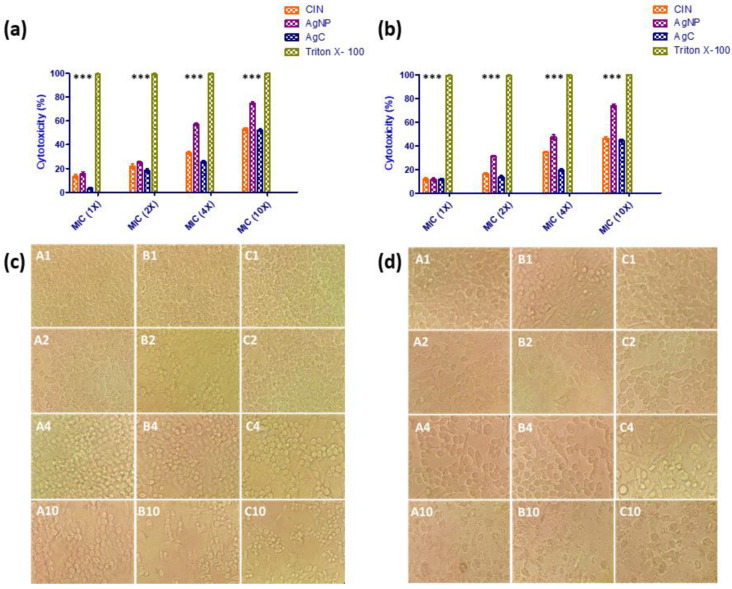
In vitro cytotoxicity of AgC. In vitro cytotoxicity of AgC on RAW 264.7 (**a**,**c**) and HEp-2 (**b**,**d**) cell lines treated with different concentrations of cinnamaldehyde (CIN) (A1-1X, A2-2X, A4-4X and A10-10XMIC), AgNPs (B1-1X, B2-2X, B4-4X and B10-10X MIC) and AgC (C1-1X, C2-2X, C4-4X and C10-10X MIC). Figure (**a**,**b**) denotes % cytotoxicity, while figure (**c**,**d**) resembles morphological changes such as loss of monolayer, vacuolization of cytoplasm, granulation in corresponding cell lines observed under microscope (40X) at 24 h of incubation. *** indicates statistically significant (*p <* 0.001) in comparison to control.

Similarly, the extent of AgC toxicity was determined using an MTT cytotoxicity assay with secondary cell lines (HEp-2 and RAW 264.7). All of the compounds tested marginally reduced the viability of secondary cell lines (RAW 264.7 and HEp-2) tested in a concentration-dependent manner. Remarkable cytotoxicity was not observed with AgC and its constituent components at lower MIC concentrations (1X, 2X, and 4X). However, at 10X MIC, RAW 264.7 and HEp-2 cells showed moderate cytotoxicity (50%) compared to cinnamaldehyde and AgNPs, which showed comparatively higher cytotoxicity (Figure 2a,b). Furthermore, at higher concentrations (10X MIC) of cinnamaldehyde, AgNPs, and AgC, typical cytopathic effects such as detachment of confluent monolayers and cytoplasmic vacuolation were observed (Figure 2c,d). In comparison to the constituent treatment compounds (cinnamaldehyde and AgNPs), AgC was found to be relatively safe and non-toxic. However, the results need to be interpreted in light of other cytotoxicity assays (including in vivo trials) before being considered as a potential therapeutic candidate.

**Figure 3:**
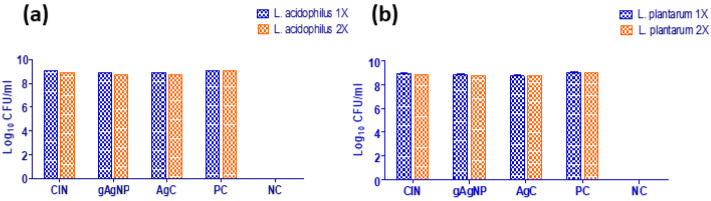
In vitro antimicrobial effect of cinnamaldehyde (CIN), AgNPs and AgC (1X and 2X) on commensal gut lactobacilli. Untreated *L. acidophilus* (**a**) and *L. plantarum* (**b**) served as positive growth control (PC), while media (MRS broth) served as negative control (NC).

Furthermore, despite treatment with AgC at 1X and 2X MIC levels, both commensal gut lactobacilli strains (*L. acidophilus* and *L. plantarum*) exhibited similar growth patterns to the control (Figure 3a,b); the inhibitory effects were non-significant, indicating that the compounds were safe against commensal gut lactobacilli.

### 3.5. In Vitro Time-and Dose-Dependent Time-Kill Kinetic Assay 

**Figure 4:**
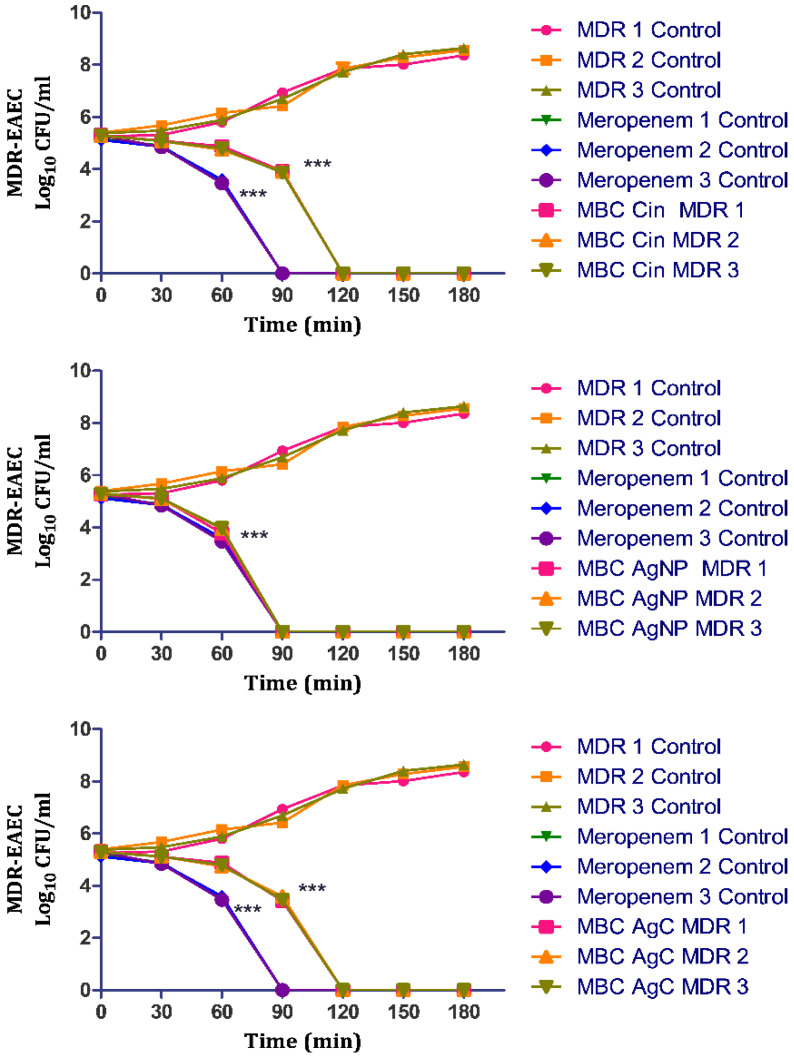
In vitro dose- and time-dependent time-kill assay of MDR-EAEC isolates co-cultured with 1X MBC of cinnamaldehyde (Cin), AgNPs and AgC in CA-MH broth at 37 °C under static conditions with respective controls of MDR-EAEC isolates (untreated and meropenem-treated). Data expressed as the mean ± standard deviation (log_10_CFU/mL) of three independent experiments (*** *p* < 0.001). Error bars too close to display.

The concentration- and time-dependent killing kinetics of AgC against MDR-EAEC strains were also investigated, with meropenem serving as the antibiotic control. In this study, the untreated bacterial control exhibited a progressively increasing growth pattern at 30, 60, 90, 120, 150 and 180 min post-co-incubation. However, the antimicrobial effect of meropenem was highly significant (*p* < 0.001) at 30 min; later, no visible growth was exhibited by the MDR-EAEC strains at 90 min post-incubation when treated with meropenem (Figure 4). The MBC levels of treatment (AgC, AgNPs, and cinnamaldehyde) exhibited highly significant (*p* < 0.001) antibacterial activity from 30 min of co-incubation. Subsequently, the MDR-EAEC strains exhibited a progressive decline in their visible growth; after 120 min of co-incubation, none of the MDR-EAEC isolates showed visible growth in any of the treatment groups. The results showed that meropenem eliminated MDR-EAEC after 90 min, while similar inhibition was observed with all other treatments, including AgC at 120 min, indicating its potent antimicrobial activity. The biological corona, providing a high negative charge on the surface of nanoparticles and a spherical shape, allows AgNPs to interact with pathogens with the highest surface area accessible, which may be one reason for their remarkable antibacterial potential [18]. Furthermore, AgNPs have been shown to cause DNA damage, mutations, and enzyme and protein inhibition [60]. This study found that AgC, which combines the properties of AgNPs and cinnamaldehyde, completely inhibited MDR-EAEC, indicating that it could be a better therapeutic alternative to antibiotics.

### 3.6. In Vivo Antibacterial Efficacy of AgC in G. mellonella Larval Model

While the study provided sufficient in vitro evidence of the antimicrobial efficacy of AgC, further in vivo efficacy testing was necessary. The larvae of *G. mellonella* were introduced as a simple, inexpensive, and quick in vivo screening model for researching a variety of microbial diseases, including EAEC, to evaluate microbial pathogenicity as well as host-pathogen interactions [25,61]. The most important feature that makes *G. mellonella* a useful pre-clinical in vivo model is its response, which shares some similarities with the mammalian innate immune system [62]. Furthermore, when compared to mammals, this mini-host has economic and ethical advantages, and its short lifespan enables it to be an ideal model for high-throughput research [27,30]. Moreover, they can easily be grown in an incubator at 37 °C, giving researchers more control over the experimental situation and allowing them to examine clinically relevant human pathogens at a temperature similar to the human host, resulting in precise and reliable data [63]. Furthermore, their survival is easily observed after the introduction of microbial diseases, and death is accompanied by unresponsiveness to bodily stimuli and extreme melanization [25]. Because the immune response of insects is a complex process that is triggered by pathogen invasion, parameters such as larvae survival, haemocyte quantification, melanization, and lactate dehydrogenase activity were estimated at various time points to study the in vivo efficacy of AgC against MDR-EAEC strains [25,64]. The determined LD_50_ of MDR-EAEC strains (1 × 10^6^ CFU/larvae), which was similar to our previous study [27], was confirmed and chosen for further experiments in this study.

#### 3.6.1. Survival Rate

**Figure 5:**
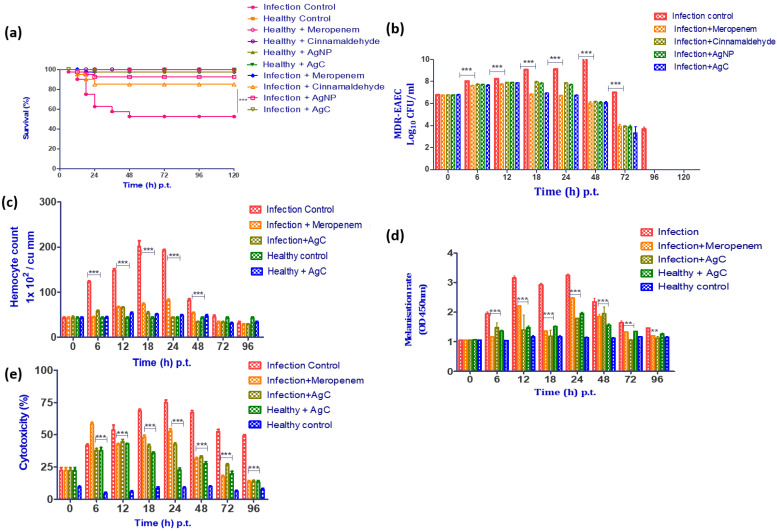
In vivo assays using *G. mellonella* model. Survival plot (**a**), MDR-EAEC counts (**b**), haemocyte density (**c**), melanization rate (**d**) and LDH cytotoxicity assay (**e**) of *G. mellonella larvae* infected with LD_50_ dose (10^6^ CFU/ larvae) of MDR-EAEC isolates treated with MBC dose of cinnamaldehyde, AgNPs and AgC 1 h p.i., keeping respective controls. MDR-EAEC counts were expressed as log_10_CFU/mL of haemolymph on EMB agar plates supplemented with ampicillin (100 μg/plate), haemocyte density as cells/mL of haemolymph, melanization rate by monitoring absorbance at 450 nm, and LDH cytotoxicity assay as the cytotoxicity (%) of larval haemolymph. Data expressed as the mean ± standard deviation of three independent experiments. ** and *** indicates (*p <* 0.01) and (*p <* 0.001), respectively and is statistically significant in comparison to infection control.

Since AgC was found to be more stable and safe in vitro with better antibacterial activity against MDR-EAEC strains, its in vivo efficacy against MDR-EAEC strains was evaluated in comparison to an effective antibiotic, meropenem. In comparison to the infection control group, treatment groups (AgC, AgNPs, cinnamaldehyde, and meropenem) had significantly higher survival rates. The bacterial infection control larval group had a survival rate of 52.50% up to 120 h p.i., whereas the meropenem-treated group had a survival rate of 97.50% (Figure 5a). While the AgNPs and cinnamaldehyde-treated infected larvae groups had significant survival rates of 92.50% and 85.0%, respectively, corresponding to a significant log-rank Mantel–Cox test (*p* < 0.001) and log-rank trend test (*p* < 0.05). The infected larval group treated with AgC outlived the treatment groups by 97.50%. Interestingly, no larval mortality was observed in uninfected control groups (PBS control, cinnamaldehyde, AgNPs, and AgC controls) up to 120 h p.t., indicating that the compounds were non-toxic to the larvae at their respective MBCs. The findings were consistent with in vitro studies that found AgC to be more stable and effective than cinnamaldehyde. The combined effects of AgNPs and cinnamaldehyde may explain the ability of AgC to protect larvae from infection by killing bacteria directly and preventing an exaggerated immune response to the disease [64,65]. The treatment compounds, on the other hand, provided varying degrees of protection to the larvae, implying that the compounds have different modes of action.

#### 3.6.2. Enumeration of MDR-EAEC Counts

When compared to the infected control group, a significant reduction (*p* < 0.001) in MDR-EAEC counts among the larval groups that were infected and treated with cinnamaldehyde, AgNPs, and AgC at 18 h p.t. (mean 1–2 log), 24 h p.t. (mean 1.2–2.3 log), and 48 h p.t. (mean 4 log) was observed (Figure 5b). This significant reduction in MDR-EAEC counts could be ascribed to the antimicrobial effects of cinnamaldehyde, AgNPs, AgC, and/or melanization metabolites [64]. In *G. mellonella* larvae, cinnamaldehyde was found to be effective in terms of better survival as well as lowering bacterial load when infected with MDR strains of *S. aureus* and *L. monocytogenes* [66,67]. The ability of cinnamaldehyde to protect the larvae has been attributed to the immune response modulation via both TRPA1-dependent and TRPA1-independent mechanisms [68]. To our surprise, AgC reduced MDR-EAEC counts more than the constituent compounds. In this study, the bacterial clearance observed at 96 h p.t. could be due to hemocyte-mediated aggregation or bacterial phagocytosis, which might have resulted in the secretion of larval AMPs, resulting in hemocyte degradation and larval melanization [25].

#### 3.6.3. Enumeration of Haemocytes

Nanoparticles are widely recognized as foreign particles that can trigger a variety of innate immune responses [69]. The amount of circulating haemocytes in *G. mellonella* larvae can fluctuate when the innate immune system is activated; therefore, the haemocyte count was determined [64,70]. In this study, the haemocyte density in the infected and treated groups of *G. mellonella* larvae increased significantly (*p* < 0.001) at 6 h p.t., peaked at 18 h p.t., and then decreased significantly (*p* < 0.001). However, no significant difference (*p* > 0.05) in haemocyte density was observed between the larval groups from 72 to 96 h p.t. (Figure 5c). The MDR-EAEC-stimulated haemocytes might have phagocytosed the bacteria during the early stages of infection, so the haemocyte density findings correlated with the bacterial enumeration assay, where no significant difference in MDR-EAEC counts was observed between the infection control group and the treatment groups. The observed reduction in circulating haemocytes in all of the studied groups at a later stage of infection could be attributed to the cytotoxic action of MDR-EAEC strains on larval cells [27]. Thus, the study demonstrated that administering AgC, AgNPs, and cinnamaldehyde increased haemocyte formation while maintaining phenoloxidase activity (determined by melanization assay) at levels comparable to uninfected larvae, thereby protecting the larvae from MDR-EAEC infection [62,64].

#### 3.6.4. Melanization Assay

The rate of melanization in the MDR-EAEC infection control larvae group was lower at 6 h p.t., then increased, peaked at 24 h p.t., and then decreased after 48 h p.t. Melanization was observed to increase in the infected group treated with AgC from 12 to 48 h p.t.; however, the intensity of melanization steadily decreased thereafter (Figure 5d). Furthermore, the NPs were found to provide larval immunomodulation, as the melanization intensity was maintained for up to 96 h p.i. in both infected and uninfected groups. The results of the melanization assay were found to be reasonably well correlated with those of the hemocyte enumeration assay, with a rise in melanization intensity detected as hemocyte density decreased. Lower levels of phenoloxidase and the presence of haemocyte nodules (which entrap and kill invading microorganisms) in response to AgC explained reduced levels of melanization in treated larvae [27,62,64]. As a result, decreasing levels of melanization in treatment groups demonstrated that AgC was safe and effective in increasing the survival of infected larvae when compared to the control.

#### 3.6.5. LDH Cytotoxicity Assay

LDH cytotoxicity in the infected control group increased significantly (*p* < 0.001) at 6 h p.t., peaked at 24 h p.t., and remained cytotoxic up to 96 h p.t. Meanwhile, there was a significant (*p* < 0.001) increase in cytotoxicity in the infected larval groups that received treatment at 6 h p.t., and the cytotoxicity remained elevated until 24 h p.t., when there was a steady decrease in cytotoxicity (Figure 5e). However, cytotoxicity was increased in uninoculated larval groups treated with meropenem, cinnamaldehyde, AgNPs, and AgC from 6 h p.t. and then gradually decreased. The infected larval group produced more LDH due to an increase in damaged and apoptotic host cells after MDR-EAEC inoculation [25]. The initial increase in LDH production in all infected groups could be explained by the early host cell damage caused by the infection before EAEC interacted with NPs. Furthermore, the slight stress caused by injecting the larvae could not be ignored as a cause of the initial LDH rise [25]. Furthermore, if AgC was toxic to the larvae, survival rates in all AgC- treated groups would have been lower. As a result of the findings, AgC was not cytotoxic to larval cells, implying that it could be used as a promising therapeutic agent.

## 4. Conclusions

This study evaluated the antimicrobial potential of biogenic AgC against MDR-EAEC field strains in a *G. mellonella* larval model. Herein, the phytochemical (cinnamaldehyde) was entrapped to AgNPs as evidenced by UV-Vis spectroscopy, FTIR, XRD, SEM, and TEM. The AgC tested was found to be stable, safe, and exerted negligible adverse effects on beneficial lactobacilli. In vivo *G. mellonella* larval studies indicated that AgC had an almost identical antibacterial potential to that of the antibiotic control (meropenem) used in this study and provided an improved immune response to the larvae against MDR-EAEC infection. Furthermore, AgC was found to be non-cytotoxic to larval cells and to have a strong immunomodulatory effect, implying that these findings were promising. 

A closer look at the proteome and genome of larvae, on the other hand, could reveal more about the host-pathogen interactions and the effect of phytocompounds and NPs on the host immune response. Furthermore, before being translated as an effective therapeutic candidate in humans or animals, AgC may be subjected to additional clinical trials in appropriate mammalian models to allow for better extrapolation of results.

## Figures and Tables

**Table 1:** Determination of MIC and MBC values of cinnamaldehyde, AgNPs and AgC against MDR-EAEC isolates.

Isolate	Cinnamaldehyde		AgNPs		AgC	
	MIC (mg/mL)	MBC (mg/mL)	MIC (mg/mL)	MBC (mg/mL)	MIC (mg/mL)	MBC (mg/mL)
MDR-1	0.256	0.512	0.008	0.016	0.008	0.008
MDR-2	0.256	0.256	0.016	0.032	0.008	0.016
MDR-3	0.512	0.512	0.016	0.032	0.016	0.032

**Table 2:** In vitro haemolytic assay of cinnamaldehyde, AgNPs and AgC in sheep RBCs.

Concentration	Haemolysis (%)		
	Cinnamaldehyde	AgNPs	AgC
MIC (1X)	3.61	0.348	0.208
MIC (2X)	4.60	0.856	0.682
MIC (5X)	9.80	1.53	0.905
MIC (10X)	12.62	2.57	3.064

## Data Availability

The data presented in this study are available on request from the corresponding author.

## Supplementary Materials

The following supporting information can be downloaded at: https://www.mdpi.com/article/10.3390/pharmaceutics14091924/s1, Table S1: Antimicrobial susceptibility pattern of EAEC strains under study; Table S2A. In vitro thermostability of cinnamaldehyde, AgNPs and AgC against MDR-EAEC isolates; Table S2B. In vitro pH stability of cinnamaldehyde, AgNPs and AgC against MDR-EAEC isolates; Table S2C. In vitro stability of cinnamaldehyde, AgNPs and AgC in physiological concentration of cationic salts against MDR-EAEC isolates; Table S2D. In vitro protease (trypsin, proteinase-K and lysozyme) stability of cinnamaldehyde, AgNPs and AgC against MDR-EAEC isolates; Table S2E. In vitro serum stability of cinnamaldehyde, AgNPs and AgC against MDR-EAEC isolates.
