# Supplementary material for: Antimicrobial Efficacy of Green Synthesized Nanosilver with Entrapped Cinnamaldehyde against Multi-Drug-Resistant Enteroaggregative Escherichia coli in Galleria mellonella
Source: Pharmaceutics. 2022 Sep 12;14(9):1924. doi: 10.3390/pharmaceutics14091924 (PMC9503582; doi:10.3390/pharmaceutics14091924)
Supplement: Supplementary file 1 [file pharmaceutics-14-01924-s001.zip › pharmaceutics-1851673-supplementary.pdf]

Supplementary Material: Antimicrobial Efficacy of Green Synthesized Nanosilver with Entrapped Cinnamaldehyde against Multi-drug-resistant Enteroaggregative *Escherichia coli* in *Galleria Mellonella*

Vemula Prasastha Ram, Jyothsna Yasur, Padikkamannil Abishad, Varsha Unni, Diksha Purushottam Gourkhede, Maria Anto Dani Nishanth, Pollumahanti Niveditha, Jess Vergis, Satya Veer Singh Malik, Byrappa Kullaiah, Nitin Vasantrao Kurkure, Chatragadda Ramesh, Laurent Dufossé, Deepak B. Rawool, Sukhadeo B. Barbuddhe

S1. *In vitro* dose and time-dependent growth kinetics of MDR-EAEC with cinnamaldehyde, AgNPs and AgC

The desired bacterial counts for each MDR-EAEC isolate, as well as cinnamaldehyde, AgNPs, and AgC were suspended in CA-MH broth as follows: Group I, 10^7 CFU of MDR- EAEC (50 μL) with 1X MBC cinnamaldehyde (50 μL); Group II, 10^7 CFU of MDR- EAEC (50 μL) with 1X MBC AgNPs; Group III, 10^7 CFU of MDR- EAEC (50 μL) with 1X MBC AgC (50 μL); Group IV, 10^7 CFU of MDR- EAEC (50 μL) with Meropenem (10 μM; 50 μL); Group V, 10^7 CFU of MDR- EAEC (50 μL) with CA-MH broth (50 μl). Similar groups were formed for the remaining two MDR-EAEC isolates. The groups, along with the appropriate controls, were incubated at 37 ^{0}C for 72 h.

S2. Enumeration of MDR-EAEC counts

G. mellonella haemolymph (n=3 larvae per group) was collected aseptically at 6 h p.t. intervals up to 24 h, then at 24 h intervals until 96 h p.t. in sterile NSS. It was thoroughly vortexed and serially diluted 10-fold in sterile NSS before being tested for the bacterial burden on EMB agar plates supplemented with 100 g of ampicillin per plate [1]. The colonies of MDR-EAEC on the plates were counted and expressed as log_{10}CFU/mL of haemolymph.

S3. Melanisation assay

G. mellonella (n=3 larvae per group) were studied at 6 h p.t. intervals for 24 h, followed by a 24 h interval until 96 h p.t. to detect melanin formation, which is an immunological marker [1]. An aliquot of the pooled haemolymph (100 L) was transferred into a 96-well microtiter plate and the optical density (OD)

at 450 nm was measured using an ELISA plate reader (Thermo Scientific Multiskan GO), with OD values from apparently healthy un-inoculated larvae serving as a background control.

S4. Enumeration of haemocytes

The haemocyte density of *G. mellonella* (n= 3 larvae per group) was evaluated by counting them in a hemocytometer chamber under a microscope at intervals of 6 h p.t. up to 24 h, followed by a 24 h interval until 96 h p.t. [1]. There was no attempt to distinguish between the various haemocyte subtypes.

S5. LDH cytotoxicity assay

G. mellonella (n=3 larvae per group) were tested for the production of LDH, a marker of cell damage, at 6 h p.t. intervals for 24 h, followed by a 24 h interval until 96 h p.t., according to the manufacturer's instructions. In this study, distilled water and 20% Triton X-100 were used as positive and negative controls, respectively. The absorbance was measured at 500 nm using an ELISA plate reader; the haemolymph from untreated larvae was used as a background control, and the cytotoxicity was calculated as Cytotoxicity (%) = $(OD_{Sample} - OD_{Control})/(OD_{Total\ Lysis} - OD_{Control}) \times 100$, wherein, the sample is the control absorbance of treated cell; control is the experimental absorbance of the untreated cell control and total lysis is the absorbance of Triton X-100 treated cells.

Table S1. **Antimicrobial susceptibility pattern of EAEC strains under study**

Isolate	Antibiotic Susceptibility testing										MIC Values (µM)								
	**CIP**	**CTR**	**AMP**	**COT**	**TE**	**IMI**	**SXT**	**GEN**	**CL**	**CO**	**CIP**	**CTR**	**AMP**	**COT**	**TE**	**IMI**	**GEN**	**CL**	**CO**
**MDR-1**	S	S	R	R	R	S	R	S	S	S	S	S	>240	>240	60	S	S	S	S
**MDR-2**	R	R	R	R	R	S	S	S	S	S	120	>240	>240	>240	2	S	S	S	S
**MDR-3**	R	R	R	R	R	S	R	S	S	S	60	>240	>240	>240	30	S	S	S	S

CIP- Ciprofloxacin, CTR- Ceftriaxone, AMP- Ampicillin, COT- Co-trimoxazole, TE- Tetracycline, IMI- Imipenem, SXT- Sulfamethoxazole, GEN- Gentamicin, CL- Chloramphenicol, CO- Colistin sulphate, S – Sensitive, R- Resistant.

Table S2A. ***In vitro*** **thermostability of cinnamaldehyde, AgNPs and AgC against MDR-EAEC isolates**

**MDR-EAEC**	**Thermostability at 70°C**								
	**Cinnamaldehyde** MIC/MBC (mg/mL)			**AgNPs** MIC/MBC (mg/mL)			**AgC** MIC/MBC (mg/mL)		
	5 min	15 min	30 min	5 min	15 min	30 min	5 min	15 min	30 min
**MDR-1**	0.512/0.512	0.512/0.512	0.512/1.024	0.008/0.008	0.008/0.008	0.008/0.008	0.008/0.008	0.008/0.008	0.008/0.008
**MDR-2**	0.512/1.024	0.512/1.024	0.1.024/1.024	0.008/0.016	0.016/0.016	0.016/0.016	0.008/0.016	0.008/0.016	0.008/0.016
**MDR-3**	1.024/1.024	1.024/1.024	0.1.024/1.024	0.008/0.016	0.008/16	0.016/0.016	0.008/0.016	0.008/0.016	0.016/0.016

**MDR-EAEC**	**Thermostability at 90°C**								
	**Cinnamaldehyde** MIC/MBC (mg/mL)			**AgNPs** MIC/MBC (mg/mL)			**AgC** MIC/MBC (mg/mL)		
	5 min	15 min	30 min	5 min	15 min	30 min	5 min	15 min	30 min
**MDR-1**	0.512/0.512	0.512/0.512	0.512/1.024	0.004/0.008	0.004/0.008	0.004/0.008	0.008/0.008	0.008/0.008	0.008/0.008
**MDR-2**	0.512/0.512	0.512/0.512	1.024/1.024	0.008/0.016	0.008/0.016	0.008/0.016	0.008/0.008	0.008/0.008	0.008/0.008
**MDR-3**	1.024/1.024	1.024/1.024	1.024/1.024	0.008/0.016	0.008/0.016	0.016/0.016	0.008/0.016	0.008/0.016	0.016/0.016

Table S2B. ***In vitro*** **pH stability of cinnamaldehyde, AgNPs and AgC against MDR-EAEC isolates**

MDR-EAEC	**Cinnamaldehyde** MIC/MBC (mg/mL)			**AgNPs** MIC/MBC (mg/mL)			**AgC** MIC/MBC (mg/mL)		
	**pH 4**	**pH 6**	**pH 8**	**pH 4**	**pH 6**	**pH 8**	**pH 4**	**pH 6**	**pH 8**
**MDR-1**	0.032/0.064	0.256/0.512	0.256/0.512	$1.25\times10^{-4}/6.25\times10^{-5}$	0.008/0.016	0.008/0.016	$1.25\times10^{-4}/6.25\times10^{-5}$	0.008/0.016	0.008/0.032
**MDR-2**	0.064/0.064	0.256/0.512	0.256/0.512	$1.25\times10^{-4}/6.25\times10^{-5}$	0.008/0.016	0.016/0.016	$2.5\times10^{-4}/6.25\times10^{-5}$	0.016/0.016	0.016/0.032
**MDR-3**	0.032/0.064	0.256/0.512	0.256/0.512	$1.25\times10^{-4}/6.25\times10^{-5}$	0.008/0.016	0.016/0.016	$2.5\times10^{-4}/6.25\times10^{-5}$	0.016/0.016	0.016/0.016

Table S2C. ***In vitro*** **stability of cinnamaldehyde, AgNPs and AgC in physiological concentration of cationic salts against MDR-EAEC isolates**

MDR-EAEC	**Cinnamaldehyde** MIC/MBC (mg/mL)		**AgNPs** MIC/MBC (mg/mL)		**AgC** MIC/MBC (mg/mL)	
	**150 mM NaCl**	**2 mM $MgCl_2$**	**150 mM NaCl**	**2 mM $MgCl_2$**	**150 mM NaCl**	**2 mM $MgCl_2$**
**MDR-1**	0.512/0.512	0.512/0.512	0.008/0.008	0.008/0.016	0.008/0.008	0.008/0.008
**MDR-2**	0.512/0.512	0.512/0.512	0.008/0.008	0.008/0.008	0.008/0.008	0.008/0.016
**MDR-3**	0.512/0.512	0.512/0.512	0.008/0.008	0.008/0.008	0.008/0.016	0.008/0.016

Table S2D. *In vitro* **protease (trypsin, proteinase-K and lysozyme) stability of cinnamaldehyde, AgNPs and AgC against MDR-EAEC isolates**

	**Trypsin**								
**MDR-EAEC**	**Cinnamaldehyde** MIC/MBC (mg/mL)			**AgNPs** MIC/MBC (mg/mL)			**AgC** MIC/MBC (mg/mL)		
	**5 min**	**15 min**	**30 min**	**5 min**	**15 min**	**30 min**	**5 min**	**15 min**	**30 min**
**MDR-1**	0.512/0.512	0.512/0.512	0.512/0.512	0.008/0.016	0.008/0.016	0.016/0.016	0.008/0.008	0.008/0.016	0.016/0.016
**MDR-2**	0.512/1.024	0.512/1.024	0.512/1.024	0.008/0.016	0.008/0.016	0.016/0.016	0.008/0.008	0.008/0.016	0.008/0.016
**MDR-3**	0.512/1.024	0.512/1.024	1.024/1.024	0.008/0.016	0.016/0.016	0.016/0.016	0.008/0.008	0.008/0.016	0.016/0.016

	**Proteinase-K**								
**MDR-EAEC**	**Cinnamaldehyde** MIC/MBC (mg/mL)			**AgNPs** MIC/MBC (mg/mL)			**AgC** MIC/MBC (mg/mL)		
	**5 min**	**15 min**	**30 min**	**5 min**	**15 min**	**30 min**	**5 min**	**15 min**	**30 min**
**MDR-1**	0.512/ 1.024	0.512/ 1.024	1.024/ 1.024	0.008/0.016	0.008/0.016	0.016/0.016	0.008/0.016	0.016/0.016	0.016/0.016
**MDR-2**	0.512/ 1.024	0.512/ 1.024	1.024/ 1.024	0.008/0.008	0.008/0.008	0.008/0.008	0.008/0.008	0.008/0.016	0.008/0.016
**MDR-3**	0.512/ 1.024	1.024/ 1.024	1.024/ 1.024	0.008/0.016	0.08/0.016	0.008/0.016	0.008/0.016	0.008/0.016	0.008/0.016

	**Lysozyme**								
**MDR-EAEC**	**Cinnamaldehyde** MIC/MBC (mg/mL)			**AgNPs** MIC/MBC (mg/mL)			**AgC** MIC/MBC (mg/mL)		
	**5 min**	**15 min**	**30 min**	**5 min**	**15 min**	**30 min**	**5 min**	**15 min**	**30 min**
**MDR-1**	0.512/ 1.024	0.512/ 1.024	0.512/ 1.024	0.008/0.016	0.016/0.016	0.016/0.016	0.008/0.008	0.008/0.008	0.008/0.008
**MDR-2**	0.512/0.512	0.512/0.512	0.512/ 1.024	0.008/0.016	0.008/0.016	0.016/0.016	0.008/0.008	0.008/0.008	0.008/0.008
**MDR-3**	0.512/ 1.024	0.512/ 1.024	0.512/ 1.024	0.008/0.016	0.016/0.016	0.016/0.016	0.008/0.008	0.008/0.016	0.016/0.016

Table S2E. ***In vitro* serum stability of cinnamaldehyde, AgNPs and AgC against MDR-EAEC isolates**

	Sheep serum								
**MDR-EAEC**	**Cinnamaldehyde** MIC/MBC (mg/mL)			**AgNPs** MIC/MBC (mg/mL)			**AgC** MIC/MBC (mg/mL)		
	**5 min**	**15 min**	**30 min**	**5 min**	**15 min**	**30 min**	**5 min**	**15 min**	**30 min**
**MDR-1**	0.512/ 1.024	1.024/ 1.024	1.024/ 1.024	0.008/0.008	0.008/0.016	0.008/16	0.008/0.008	0.008/0.008	0.008/0.008
**MDR-2**	0.512/ 1.024	1.024/ 1.024	1.024/ 1.024	0.008/0.016	0.008/0.016	0.016/0.016	0.008/0.008	0.008/0.008	0.008/0.016
**MDR-3**	0.512/0.512	0.512/0.512	0.512/0.512	0.008/0.008	0.008/0.008	0.008/0.016	0.008/0.008	0.008/0.008	0.008/0.016

	Poultry serum								
**MDR-EAEC**	**Cinnamaldehyde** MIC/MBC (mg/mL)			**AgNPs** MIC/MBC (mg/mL)			**AgC** MIC/MBC (mg/mL)		
	**5 min**	**15 min**	**30 min**	**5 min**	**15 min**	**30 min**	**5 min**	**15 min**	**30 min**
**MDR-1**	1.024/ 1.024	1.024/ 1.024	1.024/ 1.024	0.016/0.016	0.016/0.016	0.016/0.016	0.008/0.016	0.016/0.016	0.016/0.016
**MDR-2**	0.512/0.512	0.512/0.512	1.024/ 1.024	0.008/0.016	0.016/0.016	0.016/0.016	0.008/0.016	0.016/0.016	0.016/0.016
**MDR-3**	1.024/ 1.024	1.024/ 1.024	1.024/ 1.024	0.016/0.016	0.016/0.016	0.016/0.016	0.016/0.016	0.016/0.016	0.016/0.016

References:

1. Vergis, J.; Malik, S.S.; Pathak, R.; Kumar, M.; Ramanjaneya, S.; Kurkure, N.V.; Barbuddhe, S.B.; Rawool, D.B. Exploiting lactoferricin (17-30) as a potential antimicrobial and antibiofilm candidate against multi-drug-resistant enteroaggregative *Escherichia coli*. *Front. Microbiol.* **2020**, *11*, 575917, doi:10.3389/fmicb.2020.575917.
